# 2,2′-[1,1′-(Decane-1,10-diyldioxy­dinitrilo)diethyl­idyne]diphenol

**DOI:** 10.1107/S1600536809029341

**Published:** 2009-07-29

**Authors:** Xiao-Qiang Wang, Jun-Feng Tong, Wen-Kui Dong, Shang-Sheng Gong, Jian-Chao Wu

**Affiliations:** a School of Chemical and Biological Engineering, Lanzhou Jiaotong University, Lanzhou 730070, People’s Republic of China

## Abstract

The salen-type bis-oxime title compound, C_26_H_36_N_2_O_4_, lies about a crystallographic inversion centre. Classical intra­molecular O—H⋯N hydrogen bonds generate two *S*(6) ring motifs. In the crystal structure, pairs of weak inter­molecular C—H⋯O hydrogen bonds link adjacent mol­ecules into an infinite one-dimensional supra­molecular structure.

## Related literature

For the strong coordination capability and diverse biological activity of Schiff bases, see: Boskovic *et al.* (2003 ▶); Koizumi *et al.* (2005 ▶); Oshiob *et al.* (2005 ▶). For the use of Schiff base derivatives to develop protein and enzyme mimics, see: Santos *et al.* (2001 ▶). For our studies of synthesis and structure of salen-type bis­oxime compounds obtained by Schiff base reactions, see: Dong *et al.* (2008*a* ▶,*b* ▶, 2009 ▶). For hydrogen bonds, see: Desiraju (1996 ▶).
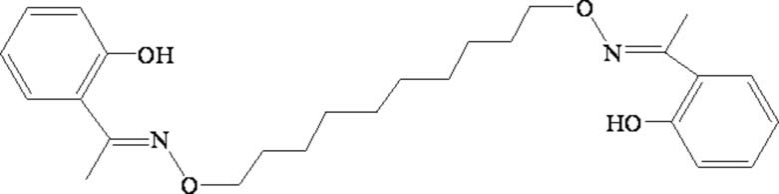


## Experimental

### 

#### Crystal data

C_26_H_36_N_2_O_4_
*M* *_r_* = 440.57
Monoclinic, 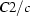

*a* = 13.0031 (16) Å
*b* = 4.6922 (6) Å
*c* = 40.654 (3) Å
β = 93.109 (2)°
*V* = 2476.8 (5) Å^3^
*Z* = 4
Mo *K*α radiation
μ = 0.08 mm^−1^
*T* = 298 K
0.50 × 0.48 × 0.23 mm

#### Data collection

Siemens SMART 1000 CCD area-detector diffractometer
Absorption correction: multi-scan (*SADABS*; Sheldrick, 1996 ▶) *T*_min_ = 0.962, *T*_max_ = 0.982
5830 measured reflections
2124 independent reflections
1209 reflections with *I* > 2σ(*I*)
*R*_int_ = 0.076

#### Refinement

*R*[*F* ^2^ > 2σ(*F* ^2^)] = 0.052
*wR*(*F* ^2^) = 0.260
*S* = 1.03
2124 reflections
145 parameters
H-atom parameters constrained
Δρ_max_ = 0.21 e Å^−3^
Δρ_min_ = −0.23 e Å^−3^

### 

Data collection: *SMART* (Siemens, 1996 ▶); cell refinement: *SAINT* (Siemens, 1996 ▶); data reduction: *SAINT*; program(s) used to solve structure: *SHELXS97* (Sheldrick, 2008 ▶); program(s) used to refine structure: *SHELXL97* (Sheldrick, 2008 ▶); molecular graphics: *SHELXTL* (Sheldrick, 2008 ▶); software used to prepare material for publication: *SHELXTL*.

## Supplementary Material

Crystal structure: contains datablocks global, I. DOI: 10.1107/S1600536809029341/ds2001sup1.cif

Structure factors: contains datablocks I. DOI: 10.1107/S1600536809029341/ds2001Isup2.hkl

Additional supplementary materials:  crystallographic information; 3D view; checkCIF report

## Figures and Tables

**Table 1:** Hydrogen-bond geometry (Å, °)

*D*—H⋯*A*	*D*—H	H⋯*A*	*D*⋯*A*	*D*—H⋯*A*
O2—H2⋯N1	0.82	1.85	2.568 (5)	146
C13—H13⋯O2^i^	0.93	2.66	3.565 (6)	163

Symmetry code: (i) 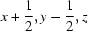.

**Fig. 1.**
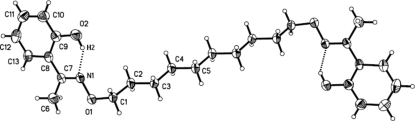
The molecular structure of the title compound with atom numbering scheme [Symmetry codes: -x + 1/2,-y + 3/2,-z + 1]. Displacement ellipsoids for non-hydrogen atoms are drawn at the 30% probability level.

**Fig. 2.**
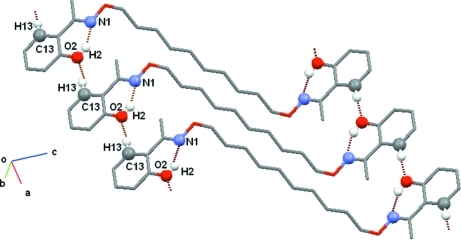
Part of the one-dimensional supramolecular structure of the title compound. Intramolecular and intermolecular hydrogen bonds of the title compound are shown as dashed lines.

## supplementary crystallographic information

### Comment

Schiff bases are one of most prevalent mixed-donor ligands in the field of coordination chemistry and have been intensively studied due to their strong coordination capability as well as their diverse biological activities, such as antibacterial, antitumor, *etc*. (Koizumi *et al.*, 2005; Boskovic *et al.*, 2003; Oshiob *et al.*, 2005). In addition, many Schiff base derivatives have been synthesized and employed to develop protein and enzyme mimics (Santos *et al.*, 2001). Our group is interested in the synthesis and structure of salen-type bisoxime compounds by Schiff base reaction (Dong *et al.*, 2008*a*; Dong *et al.*, 2008*b*). Here, we report, for the first time, the synthesis and crystal structure of a salen-type bisoxime compound containing ten-methene bridge, 2,2'-[1,1'-(decane-1,10-diyldioxydinitrilo)diethylidyne]diphenol.

Perspective view of the title molecule, showing the atomic numbering scheme, is given in Fig. 1. Each molecule exists in a *trans* configuration with respect to the methylidene unit. The two phenyl rings in each molecule are parallel to each other, with C1—O1—N1—C7 torsion angles of 178.7 (3)° and a perpendicular interplanar spacing of *ca* 6.695 (2) Å. In each title compound, there exist two classical intramolecular O—H···N hydrogen bonds (Fig. 1) which generate two six-membered rings, producing two S(6) ring motifs. In the crystal structure, pairs of weak intermolecular C—H···O hydrogen bonds (Table 1, Fig. 2)(Desiraju, 1996) link the adjacent molecules into an infinite one-dimensional supramolecular structure (Fig. 2).

### Experimental

2,2'-[1,1'-(Decane-1,10-diyldioxydinitrilo)diethylidyne]diphenol was synthesized according to the literature (Dong, *et al.*, 2009). To an absolute ethanol solution (4 ml) of 2'-hydroxyacetophenone (375.8 mg, 2.76 mmol) was added an absolute ethanol solution (4 ml) of 1, 10-bis(aminooxy)decane (180.9 mg, 1.38 mmol). The mixture solution was stirred at 328–333 K for 48 h. When cool to room temperature (298 K), white precipitate was formed which was filtered and washed successively with absolute ethanol (2 ml) and n-hexane (8 ml), respectively. The product was dried under vacuum and purified by recrystallization from ethanol to yield 331.9 mg of the title compound. Yield, 52.75%. m. p. 343–344 K. Anal. Calcd. for C_26_H_36_N_2_O_4_: C, 70.88; H, 8.24; N, 6.36. Found: C, 70.59; H, 8.23; N, 6.57.

Colorless block-like single crystals suitable for X-ray diffraction studies were obtained after several days by slow evaporation from a diethyl ether solution of the title compound.

### Refinement

Non-H atoms were refined anisotropically. H atoms were treated as riding atoms with distances C—H = 0.96 Å (CH_3_), 0.97 Å (CH_2_), 0.93 Å (CH), 0.82 Å (OH), and*U*_iso_(H) = 1.20 *U*_eq_(C) for methylene and methylidyne, 1.50 *U*_eq_(C) for methyl, 1.50 *U*_eq_(O). The crystal quality was not good enough to provide data completeness to 1.0, which is also reflected in the weighted *R*.

### Crystal data

**Table:**

C_26_H_36_N_2_O_4_	*F*(000) = 952
*M**_r_* = 440.57	*D*_x_ = 1.181 Mg m^−^^3^
Monoclinic, *C*2/*c*	Melting point = 343–344 K
Hall symbol: -C 2yc	Mo *K*α radiation, λ = 0.71073 Å
*a* = 13.0031 (16) Å	Cell parameters from 1361 reflections
*b* = 4.6922 (6) Å	θ = 2.5–26.9°
*c* = 40.654 (3) Å	µ = 0.08 mm^−^^1^
β = 93.109 (2)°	*T* = 298 K
*V* = 2476.8 (5) Å^3^	Block-like, colorless
*Z* = 4	0.50 × 0.48 × 0.23 mm

### Data collection

**Table:**

Siemens SMART 1000 CCD area-detector diffractometer	2124 independent reflections
Radiation source: fine-focus sealed tube	1209 reflections with *I* > 2σ(*I*)
graphite	*R*_int_ = 0.076
φ and ω scans	θ_max_ = 25.0°, θ_min_ = 2.0°
Absorption correction: multi-scan (*SADABS*; Sheldrick, 1996)	*h* = −15→15
*T*_min_ = 0.962, *T*_max_ = 0.982	*k* = −5→5
5830 measured reflections	*l* = −44→48

### Refinement

**Table:**

Refinement on *F*^2^	Primary atom site location: structure-invariant direct methods
Least-squares matrix: full	Secondary atom site location: difference Fourier map
*R*[*F*^2^ > 2σ(*F*^2^)] = 0.052	Hydrogen site location: inferred from neighbouring sites
*wR*(*F*^2^) = 0.260	H-atom parameters constrained
*S* = 1.03	*w* = 1/[σ^2^(*F*_o_^2^) + (0.1008*P*)^2^ + 5.3906*P*] where *P* = (*F*_o_^2^ + 2*F*_c_^2^)/3
2124 reflections	(Δ/σ)_max_ < 0.001
145 parameters	Δρ_max_ = 0.21 e Å^−^^3^
0 restraints	Δρ_min_ = −0.23 e Å^−^^3^

### Special details

**Table:**

Geometry. All e.s.d.'s (except the e.s.d. in the dihedral angle between two l.s. planes) are estimated using the full covariance matrix. The cell e.s.d.'s are taken into account individually in the estimation of e.s.d.'s in distances, angles and torsion angles; correlations between e.s.d.'s in cell parameters are only used when they are defined by crystal symmetry. An approximate (isotropic) treatment of cell e.s.d.'s is used for estimating e.s.d.'s involving l.s. planes.
Refinement. Refinement of *F*^2^ against ALL reflections. The weighted *R*-factor *wR* and goodness of fit *S* are based on *F*^2^, conventional *R*-factors *R* are based on *F*, with *F* set to zero for negative *F*^2^. The threshold expression of *F*^2^ > σ(*F*^2^) is used only for calculating *R*-factors(gt) *etc*. and is not relevant to the choice of reflections for refinement. *R*-factors based on *F*^2^ are statistically about twice as large as those based on *F*, and *R*- factors based on ALL data will be even larger.

### Fractional atomic coordinates and isotropic or equivalent isotropic displacement parameters (Å^2^)

**Table:**

	*x*	*y*	*z*	*U*_iso_*/*U*_eq_	
O1	0.6948 (2)	0.6651 (8)	0.60162 (7)	0.0693 (9)	
O2	0.5126 (2)	0.3324 (9)	0.66379 (8)	0.0803 (11)	
H2	0.5375	0.4228	0.6489	0.120*	
N1	0.6559 (2)	0.5081 (8)	0.62776 (8)	0.0544 (9)	
C1	0.6125 (3)	0.8304 (11)	0.58630 (10)	0.0620 (12)	
H1A	0.6413	0.9723	0.5721	0.074*	
H1B	0.5769	0.9302	0.6032	0.074*	
C2	0.5353 (3)	0.6509 (11)	0.56611 (10)	0.0573 (11)	
H2A	0.5718	0.5358	0.5507	0.069*	
H2B	0.5010	0.5226	0.5807	0.069*	
C3	0.4551 (3)	0.8281 (11)	0.54718 (10)	0.0584 (11)	
H3A	0.4895	0.9470	0.5316	0.070*	
H3B	0.4227	0.9536	0.5625	0.070*	
C4	0.3718 (3)	0.6584 (11)	0.52849 (10)	0.0596 (11)	
H4A	0.3377	0.5384	0.5440	0.071*	
H4B	0.4040	0.5342	0.5130	0.071*	
C5	0.2911 (3)	0.8377 (11)	0.50978 (10)	0.0639 (12)	
H5A	0.3253	0.9613	0.4947	0.077*	
H5B	0.2574	0.9581	0.5253	0.077*	
C6	0.8336 (3)	0.3275 (14)	0.63241 (13)	0.0859 (17)	
H6A	0.8384	0.1795	0.6163	0.129*	
H6B	0.8786	0.2842	0.6513	0.129*	
H6C	0.8535	0.5060	0.6231	0.129*	
C7	0.7246 (3)	0.3474 (10)	0.64272 (9)	0.0508 (10)	
C8	0.6903 (3)	0.1753 (10)	0.67002 (9)	0.0510 (10)	
C9	0.5870 (3)	0.1717 (11)	0.67948 (11)	0.0626 (12)	
C10	0.5576 (4)	0.0034 (13)	0.70503 (12)	0.0759 (14)	
H10	0.4895	0.0057	0.7109	0.091*	
C11	0.6286 (4)	−0.1700 (13)	0.72215 (12)	0.0785 (15)	
H11	0.6078	−0.2850	0.7392	0.094*	
C12	0.7295 (4)	−0.1711 (12)	0.71384 (12)	0.0778 (15)	
H12	0.7773	−0.2859	0.7254	0.093*	
C13	0.7603 (3)	−0.0015 (11)	0.68828 (10)	0.0639 (12)	
H13	0.8290	−0.0041	0.6830	0.077*	

### Atomic displacement parameters (Å^2^)

**Table:**

	*U*^11^	*U*^22^	*U*^33^	*U*^12^	*U*^13^	*U*^23^
O1	0.0520 (16)	0.081 (2)	0.0734 (19)	−0.0094 (18)	−0.0129 (14)	0.0220 (19)
O2	0.0520 (17)	0.085 (2)	0.104 (2)	0.0082 (19)	0.0014 (16)	0.027 (2)
N1	0.0458 (17)	0.058 (2)	0.0580 (19)	−0.0004 (19)	−0.0087 (15)	0.0059 (19)
C1	0.057 (2)	0.064 (3)	0.064 (2)	−0.010 (3)	−0.016 (2)	0.019 (2)
C2	0.051 (2)	0.061 (3)	0.058 (2)	0.004 (2)	−0.0098 (18)	0.009 (2)
C3	0.055 (2)	0.060 (3)	0.060 (2)	−0.003 (2)	−0.0069 (19)	0.017 (2)
C4	0.053 (2)	0.062 (3)	0.063 (2)	−0.001 (3)	−0.0090 (19)	0.012 (2)
C5	0.054 (2)	0.071 (3)	0.066 (3)	0.003 (3)	−0.011 (2)	0.016 (3)
C6	0.056 (3)	0.100 (4)	0.102 (4)	0.008 (3)	0.001 (3)	0.029 (4)
C7	0.045 (2)	0.050 (2)	0.056 (2)	0.002 (2)	−0.0076 (18)	−0.007 (2)
C8	0.051 (2)	0.046 (2)	0.055 (2)	0.012 (2)	−0.0067 (18)	−0.007 (2)
C9	0.062 (3)	0.056 (3)	0.069 (3)	0.005 (3)	0.000 (2)	−0.002 (3)
C10	0.074 (3)	0.075 (3)	0.079 (3)	0.003 (3)	0.010 (3)	0.003 (3)
C11	0.102 (4)	0.066 (3)	0.067 (3)	−0.003 (4)	0.008 (3)	0.006 (3)
C12	0.101 (4)	0.065 (3)	0.066 (3)	0.024 (3)	−0.010 (3)	−0.002 (3)
C13	0.066 (3)	0.062 (3)	0.062 (3)	0.013 (3)	−0.007 (2)	−0.005 (3)

### Geometric parameters (Å, °)

**Table:**

O1—N1	1.409 (4)	C5—H5A	0.9700
O1—C1	1.436 (5)	C5—H5B	0.9700
O2—C9	1.359 (5)	C6—C7	1.502 (6)
O2—H2	0.8200	C6—H6A	0.9600
N1—C7	1.296 (5)	C6—H6B	0.9600
C1—C2	1.517 (6)	C6—H6C	0.9600
C1—H1A	0.9700	C7—C8	1.462 (6)
C1—H1B	0.9700	C8—C13	1.413 (5)
C2—C3	1.511 (5)	C8—C9	1.417 (6)
C2—H2A	0.9700	C9—C10	1.376 (7)
C2—H2B	0.9700	C10—C11	1.389 (7)
C3—C4	1.515 (6)	C10—H10	0.9300
C3—H3A	0.9700	C11—C12	1.373 (7)
C3—H3B	0.9700	C11—H11	0.9300
C4—C5	1.516 (5)	C12—C13	1.385 (7)
C4—H4A	0.9700	C12—H12	0.9300
C4—H4B	0.9700	C13—H13	0.9300
C5—C5^i^	1.537 (8)		
			
N1—O1—C1	108.7 (3)	C4—C5—H5B	108.8
C9—O2—H2	109.5	C5^i^—C5—H5B	108.8
C7—N1—O1	113.1 (3)	H5A—C5—H5B	107.7
O1—C1—C2	113.0 (4)	C7—C6—H6A	109.5
O1—C1—H1A	109.0	C7—C6—H6B	109.5
C2—C1—H1A	109.0	H6A—C6—H6B	109.5
O1—C1—H1B	109.0	C7—C6—H6C	109.5
C2—C1—H1B	109.0	H6A—C6—H6C	109.5
H1A—C1—H1B	107.8	H6B—C6—H6C	109.5
C3—C2—C1	112.8 (4)	N1—C7—C8	116.5 (3)
C3—C2—H2A	109.0	N1—C7—C6	122.8 (4)
C1—C2—H2A	109.0	C8—C7—C6	120.6 (4)
C3—C2—H2B	109.0	C13—C8—C9	116.4 (4)
C1—C2—H2B	109.0	C13—C8—C7	120.6 (4)
H2A—C2—H2B	107.8	C9—C8—C7	123.1 (4)
C2—C3—C4	114.9 (4)	O2—C9—C10	116.9 (4)
C2—C3—H3A	108.5	O2—C9—C8	121.9 (4)
C4—C3—H3A	108.5	C10—C9—C8	121.2 (4)
C2—C3—H3B	108.5	C9—C10—C11	120.7 (5)
C4—C3—H3B	108.5	C9—C10—H10	119.7
H3A—C3—H3B	107.5	C11—C10—H10	119.7
C3—C4—C5	114.6 (4)	C12—C11—C10	119.8 (5)
C3—C4—H4A	108.6	C12—C11—H11	120.1
C5—C4—H4A	108.6	C10—C11—H11	120.1
C3—C4—H4B	108.6	C11—C12—C13	120.1 (5)
C5—C4—H4B	108.6	C11—C12—H12	119.9
H4A—C4—H4B	107.6	C13—C12—H12	119.9
C4—C5—C5^i^	113.9 (5)	C12—C13—C8	121.8 (4)
C4—C5—H5A	108.8	C12—C13—H13	119.1
C5^i^—C5—H5A	108.8	C8—C13—H13	119.1
			
C1—O1—N1—C7	178.7 (3)	C13—C8—C9—O2	−180.0 (4)
N1—O1—C1—C2	−72.6 (4)	C7—C8—C9—O2	−0.7 (7)
O1—C1—C2—C3	−174.0 (4)	C13—C8—C9—C10	−0.2 (7)
C1—C2—C3—C4	−175.9 (4)	C7—C8—C9—C10	179.0 (4)
C2—C3—C4—C5	179.5 (4)	O2—C9—C10—C11	179.3 (5)
C3—C4—C5—C5^i^	178.5 (5)	C8—C9—C10—C11	−0.5 (8)
O1—N1—C7—C8	−179.5 (3)	C9—C10—C11—C12	0.9 (8)
O1—N1—C7—C6	−1.4 (6)	C10—C11—C12—C13	−0.5 (8)
N1—C7—C8—C13	−178.6 (4)	C11—C12—C13—C8	−0.2 (8)
C6—C7—C8—C13	3.3 (6)	C9—C8—C13—C12	0.6 (6)
N1—C7—C8—C9	2.2 (6)	C7—C8—C13—C12	−178.7 (4)
C6—C7—C8—C9	−175.9 (5)		

Symmetry codes: (i) −*x*+1/2, −*y*+3/2, −*z*+1.

### Hydrogen-bond geometry (Å, °)

**Table:**

*D*—H···*A*	*D*—H	H···*A*	*D*···*A*	*D*—H···*A*
O2—H2···N1	0.82	1.85	2.568 (5)	146
C13—H13···O2^ii^	0.93	2.66	3.565 (6)	163

Symmetry codes: (ii) *x*+1/2, *y*−1/2, *z*.
